# Successful Stabilization of Thyroid Storm Precipitated by Medication Noncompliance and Complicated Urinary Tract Infection: A Case Report

**DOI:** 10.7759/cureus.21048

**Published:** 2022-01-09

**Authors:** Nicole K Hunzeker, Abid Choudhury

**Affiliations:** 1 Internal Medicine, Jamaica Hospital Medical Center, New York, USA

**Keywords:** hyperbilirubinemia, graves´disease, medication noncompliance, uti, thyrotoxicosis, hyperthyroidism, thyroid storm

## Abstract

Thyroid storm is a rare but life-threatening condition that can occur in the setting of incompletely treated or untreated hyperthyroidism, and is often precipitated by recent infection, surgery, or trauma. We present a fascinating case report of the successful stabilization of a 29-year-old female, recently diagnosed with hyperthyroidism and non-compliant with medications, who presented with thyroid storm in the setting of sepsis secondary to right-sided pyelonephritis, non-ST-segment elevation myocardial infarction (NSTEMI) type 2, and hyperbilirubinemia. Management was further complicated by electrolyte imbalances secondary to severe diarrhea due to the thyroid storm and pancytopenia likely due to aggressive hydration. This case is demonstrative of the importance of early recognition, timely management, and patient education of life-threatening endocrine disorders such as severe thyrotoxicosis.

## Introduction

Thyroid storm is a rare but life-threatening condition with an incidence estimated between 5.7 and 7.6 cases/1 million US persons per year [1]. This complication of hyperthyroidism occurs in the context of hyperthyroidism that is incompletely treated or untreated, and it is often precipitated by recent infection, surgery, or trauma. Manifestations of thyroid storm include fever, diaphoresis, altered mental status, gastrointestinal-hepatic dysfunction, and cardiovascular dysfunction such as tachyarrhythmias, which may eventually result in cardiovascular collapse and death. Early recognition and treatment of thyroid storm is imperative, as the condition carries a mortality rate of 10-20% [2,3].

## Case presentation

A 29-year-old female with a past medical history of hyperthyroidism was brought in by Emergency Medical Services for fever, headache, and palpitations of one-day duration. Her symptoms were not alleviated or exacerbated by anything. She recently had a urinary tract infection (UTI) three weeks ago that was treated with a full course of antibiotics. The patient was admitted with severe chills, malaise, and diarrhea. She denied dysuria, increased urinary frequency, chest pain, shortness of breath, loss of consciousness, and neck stiffness. Her past medical history was significant for recent UTI, gastroesophageal reflux disease, recently diagnosed hyperthyroidism, and vaginal bleeding during pregnancy. There is no history of recent travel and no known sick contacts. She denies use of alcohol, tobacco, and recreational drugs. She denies any significant family history. Surgical history is significant for one cesarean-section. Her medication usage consists of occasional ibuprofen. She was recently diagnosed with hyperthyroidism, and admits to taking the medication that she was prescribed for five days, but then self-discontinued the medication. 

**Figure 1:**
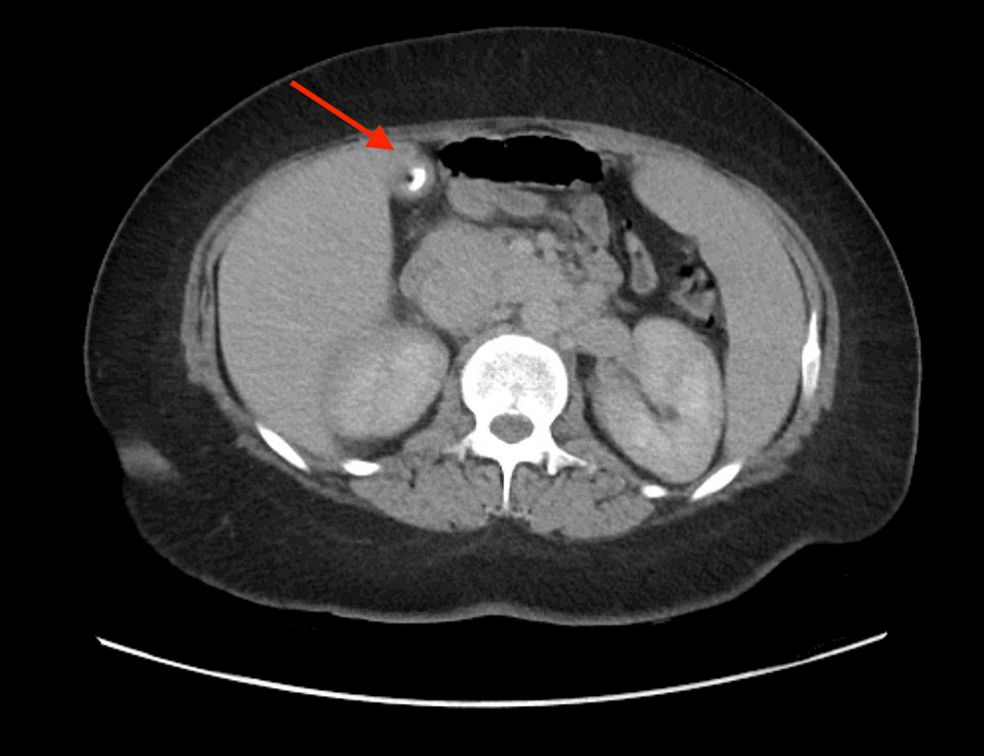
CT abdomen and pelvis CT, computed tomography. Arrow delineates calcified gallstones without obstruction.

**Figure 2:**
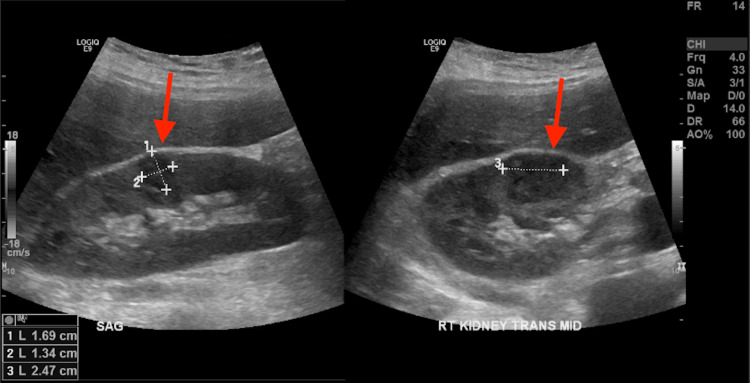
Right upper quadrant ultrasound Arrows delineate slight parenchymal heterogeneity in the upper and mid right kidney, consistent with pyelonephritis.

**Figure 3:**
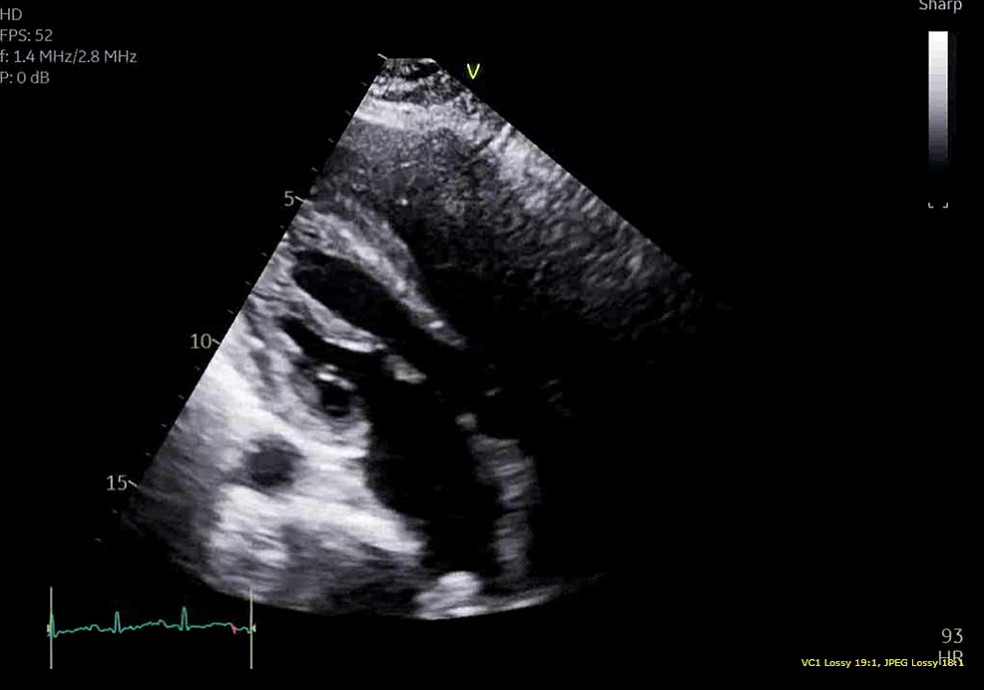
Transthoracic echocardiogram Findings of transthoracic echocardiogram consistent with hyperdynamic systolic dysfunction and increased flow across the left ventricular outflow tract.

**Figure 4:**
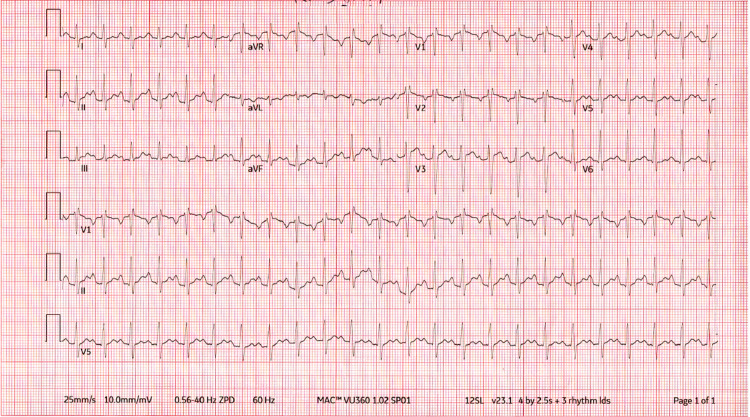
Initial electrocardiogram Initial electrocardiogram demonstrated sinus tachycardia and nonspecific ST-T wave changes.

Initial vitals in the emergency room showed a blood pressure of 118/84 mm Hg, pulse of 156 bpm, 103°F temperature, respiration rate of 17 breaths/min, and SpO_2_ of 98% on room air. Physical examination was significant for an obese, ill-appearing, diaphoretic woman appearing her stated age with tachycardia and diffuse, nontender thyromegaly. Initial laboratory findings are outlined in Table 1. Urinalysis was grossly positive for UTI with large blood, positive nitrites. Urine toxicology was negative. The patient was then sent for CT abdomen and pelvis (Figure 1) and ultrasound of the abdominal right upper quadrant (Figure 2), which showed findings consistent with right-sided pyelonephritis, cystitis, cholelithiasis without obstruction, and hepatomegaly. The patient was also sent for transthoracic echocardiogram (Figure 3), which showed hyperdynamic systolic dysfunction and increased flow across the left ventricular outflow tract. No prior imaging was available for comparison. Initial electrocardiogram (EKG) revealed sinus tachycardia and nonspecific ST-T wave changes (Figure 4). Blood and urine cultures were sent. The patient was given 3 liters of intravenous normal saline, doses of vancomycin and cefepime, and admitted for thyroid storm and UTI.

**Table 1:** Summary of the results of laboratory findings PTT, partial thromboplastin time; PT, prothrombin time; INR, international normalized ratio; TSH, thyroid-stimulating hormone.

Laboratory Investigation	Reference Range	Patient's Value on Admission	Patient's Value 2 Years Ago
Hemoglobin	13.0-18.0 g/dL	10.1	11.2
White Blood Cell	4.0-11.0/mL	17.9	10.1
Platelet	140-450 /mL	156	280
Lactate	0.00-2.00 mmol/L	0.84	
Troponin	<0.04 ng/dL	0.07	
Total Bilirubin	0.2-1.2 mg/dL	3.3	1.1
Bilirubin, Direct	0.0-0.3 mg/dL	0.4	
Bilirubin, Indirect	0.2-0.8 mg/dL	2.9	
Albumin	3.4-5.0 g/dL	3.7	4.3
Alkaline Phosphatase	46-116 U/L	231	106
Lipase	23-300 U/L	22	
Alanine Transferase	14-63 U/L	20	19
Aspartate Transferase	15-37 U/L	30	25
Blood Urea Nitrogen	7-18 mg/dL	12	10
Glucose	<100 mg/dL	91	80
Creatinine	0.7-1.3 mg/dL	0.4	0.5
Sodium	136-145 mEQ/L	136	140
Potassium	3.5-5.1 mEQ/L	3.5	4.9
Chloride	98-107 mEQ/L	105	100
Calcium	8.4-10.2 mg/dL	9.1	9.5
PTT	27.6-37.5 seconds	37.2	
PT	10.8-12.9 seconds	19.1	
INR	0.9-1.1	1.5	
TSH	0.400-4.000 mIU/mL	<0.05	0.58
Free T4	0.71-1.9 ng/dL	>6.99	
T3	80-200 ng/dL	>22.8	
Erythrocyte Sedimentation Rate	0-20 mm/h	95	
C-Reactive Protein	0.3-10.0 mg/dL	7.9	

Once admitted, endocrinology was consulted for the management of thyroid storm. Endocrinology recommended starting the patient on metoprolol 50 mg PO every 12 hours, hydrocortisone 100 mg IV every 8 hours, and methimazole 20 mg PO every 6 hours. Endocrinology also ordered thyroid-stimulating hormone (TSH) receptor antibody testing, which later resulted >40.00, consistent with thyroid storm due to Grave’s disease precipitated by medication noncompliance and recent UTI.

**Figure 5:**
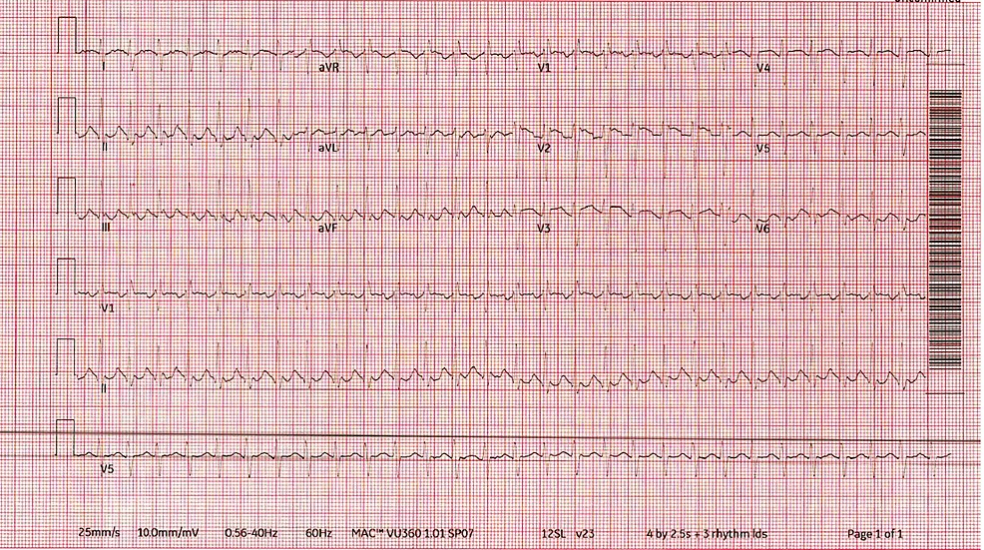
Repeat electrocardiogram Repeat electrocardiogram showing rightward axis deviation and nonspecific ST and T wave abnormalities.

Additional labs revealed an upward-trending troponin, and repeat EKG showed rightward axis deviation and nonspecific ST and T wave abnormalities (Figure 5). These findings in the setting of thyroid storm are consistent with non-ST-segment elevation myocardial infarction (NSTEMI) type 2 likely due to supply/demand mismatch from septic state and hypermetabolic state from the thyroid storm.

On hospital day 2, the patient began complaining of multiple episodes of nonbloody, nonmucoid loose stools. Repeat basic metabolic panel showed a low potassium level of 2.8, so the patient was given potassium and magnesium replenishment, and levels normalized. Repeat complete blood count showed pancytopenia (leukopenia 3.6, microcytic anemia 7.7, thrombocytopenia 125), which was likely unmasked by the rehydration therapy she was given. The leukopenia specifically was likely due in part to the side effect profile of methimazole. The patient’s lab values continued to be closely monitored. On hospital day 3, the patient had a heart rate of 200 bpm. After additional doses of metoprolol, it later stabilized to around 80 bpm. The patient’s blood culture resulted negative and urine culture resulted positive for *Escherichia coli* at this time, so her antibiotic regime was switched to cefazolin 2 g IV every 8 hours. 

On the fourth day of hospitalization, the patient was transferred from the intensive care unit to the regular floor. At this time, the patient wished to sign out against medical advice. The patient was oriented to person, place, time, and situation, and was able to verbalize why she was admitted, and what could happen if she leaves against medical advice. She agreed to take her medications as prescribed and to follow up with her primary care physician and endocrinology outpatient.

## Discussion

There is no formal criterion for diagnosing thyroid storm, but clinical presentation in tandem with the depressed TSH levels and elevated T3/T4 are used to support the diagnosis. Although it is not the definitive diagnostic criterion, a scoring system was developed by Burch and Wartofsky [4] that assigns numeric values to clinical symptoms such as degree of fever, cardiovascular dysfunction, and gastrointestinal-hepatic dysfunction (Table 2). A score of 45 or greater is highly sensitive for thyroid storm. Following this criterion, this patient received a score of 70 (25 points for fever of 103-103.9, 10 points for diarrhea, 25 points for tachycardia >140 bpm, and 10 points for positive precipitant history), consistent with thyroid storm.

**Table 2:** Burch and Wartofsky diagnostic criteria for thyroid storm Score of ≥45 or more is highly suggestive of thyroid storm [4].

Thermoregulatory Dysfunction	
Temperature	Points
99-99.9°F	5
100-100.9°F	10
101-101.9°F	15
102-102.9°F	20
103-103.9°F	25
≥104.0°F	30
Central Nervous System Effects	
Mild - Agitation	10
Moderate - Delirium, Psychosis, Extreme Lethargy	20
Severe - Seizure, Coma	30
Gastrointestinal-Hepatic Dysfunction	
Moderate - Diarrhea, Nausea/Vomiting, Abdominal Pain	10
Severe - Unexplained Jaundice	20
Cardiovascular Dysfunction	
Tachycardia	5
99-109	10
110-119	15
120-129	20
130-139	25
≥140	30
Atrial Fibrillation	10
Heart Failure	
Mild - Pedal Edema	5
Moderate - Bibasilar Rales	10
Severe - Pulmonary Edema	15
Precipitant History	
Negative	0
Positive	10

In addition to the diagnosis of thyroid storm being quite rare, this case garners additional interest through the complex comorbidities and patient education issues present. For example, as a result of the aggressive IV hydration the patient was treated with, she ended up developing a dilutional pancytopenia. Additionally, the leukopenia that she developed was likely a result of a side effect of methimazole. In an ideal scenario, this patient would not have signed out against medical advice so that her labs could be monitored very closely. Given these less-than-ideal circumstances, the patient will need to have her blood work followed up at her outpatient appointments to assess her thyroid levels, the status of her pancytopenia, and her electrolyte levels. An additional comorbidity in this particular case was discovered through the abnormal EKG and elevated troponin levels, suggesting NSTEMI type 2 as a result of supply/demand mismatch and hypermetabolic state as a result of the thyroid storm.

Many previous case reports of thyroid storm in young women have delineated cases occurring in the setting of pregnancy, labor, during cesarean-section, and postpartum [5-7]. This case report differs from previous case reports in that we present a case of thyroid storm in a young woman occurring outside these contexts and instead in the context of a UTI and medication noncompliance. It is evident that this case report offers a unique vignette of thyroid storm in a nonpregnant, nonpostpartum young woman. 

Additionally, this case report is unique from previous case reports regarding the topic of thyroid storm in that this case occurred in previously diagnosed hyperthyroidism in the setting of medication noncompliance. Many previous reports document thyroid storm occurring as the initial presentation of undiagnosed underlying hyperthyroidism [5,8,9]. Our patient had already been diagnosed with hyperthyroidism, and her thyroid storm could have been prevented by adherence to her medication. This case demonstrates why it is not only important but necessary to educate patients regarding their medications. This case raises very interesting themes surrounding patient education. This patient did not understand the potential complications surrounding a diagnosis of hyperthyroidism, and stated that she did not perceive the condition as life threatening. The patient did not possess a clear understanding of the importance of medication compliance, as evidenced by her self-discontinuing her medications after only five days of taking them for the often-cited reason of not feeling like she needed them. Had the patient been properly informed about the adverse effects of ceasing her medication, it likely would have deterred her from self-discontinuing the medication. There is substantial value in educating our patients that prescribed medications should be taken as prescribed, even when the patient is feeling well. We classically see this issue frequently with patients self-discontinuing an antibiotic before the prescribed course is complete, as well as in patients with asymptomatic hypertension. This case is an important reminder to always discuss and assess for patient understanding of why the medication is being prescribed and the importance of taking the medication as prescribed, because doing so can ultimately positively impact patient outcomes and help avoid life-threatening complications.

## Conclusions

Thyroid storm is a life-threatening condition that, although rare, clinicians should maintain a high degree of suspicion for in the context of thermoregulatory dysfunction, central nervous system effects, gastrointestinal-hepatic dysfunction, cardiovascular dysfunction, heart failure, and precipitant history. Physicians should hold a high degree of clinical suspicion for thyroid storm in the setting of untreated or incompletely treated hyperthyroidism, infection, trauma, or surgery. Early recognition allows for the best medical management optimization of the thyroid storm and any existing comorbidities. Although rare, thyroid storm is a life-threatening complication of hyperthyroidism, and all patients diagnosed with hyperthyroidism deserve to be educated on this potential risk and to be informed of the importance of taking their medications as prescribed.

## References

[1] Galindo RJ, Hurtado CR, Pasquel FJ, García Tome R, Peng L, Umpierrez GE (2019). National trends in incidence, mortality, and clinical outcomes of patients hospitalized for thyrotoxicosis with and without thyroid storm in the United States, 2004-2013. Thyroid.

[2] Chiha M, Samarasinghe S, Kabaker AS (2015). Thyroid storm: an updated review. J Intensive Care Med.

[3] Carroll R, Matfin G (2010). Endocrine and metabolic emergencies: thyroid storm. Ther Adv Endocrinol Metab.

[4] Burch HB, Wartofsky L (1993). Life-threatening thyrotoxicosis. Thyroid storm. Endocrinol Metab Clin North Am.

[5] Ma Y, Li H, Liu J, Lin X, Liu H (2018). Impending thyroid storm in a pregnant woman with undiagnosed hyperthyroidism: a case report and literature review. Medicine (Baltimore).

[6] Saleem M, Sethi SM, Ali A, Kiran Z (2021). Metastatic choriocarcinoma in a young woman presenting as thyroid storm: a case report. J Med Case Rep.

[7] Sugiyama Y, Tanaka R, Yoshiyama Y (2017). A case of sudden onset of thyroid storm just before cesarean section manifesting congestive heart failure and pulmonary edema. JA Clin Rep.

[8] Hosalli N, Ravishankar Ravishankar, Ramesh SS, Balagi V (2020). Thyroid storm presenting as jaundice - a rare case report. J Assoc Physicians India.

[9] Yamazaki K, Minakata K, Nakane T, Kawatou M, Minatoya K, Sakata R (2021). Thyroid storm after mitral valve repair in a patient with Becker muscular dystrophy. J Card Surg.

